# Research on the Electrochemical Impedance Spectroscopy Evolution of Sodium-Ion Batteries in Different States

**DOI:** 10.3390/molecules29204963

**Published:** 2024-10-20

**Authors:** Xiong Shu, Yongjing Li, Bowen Yang, Qiong Wang, Konlayutt Punyawudho

**Affiliations:** 1 Hunan Provincial Key Laboratory of Vehicle Power and Transmission System, Hunan Institute of Engineering, Xiangtan 411104, China; 19967298952@163.com (Y.L.);; 2 Department of Mechanical Engineering, Chiang Mai University, Chiang Mai 50200, Thailand

**Keywords:** sodium-ion battery, electric vehicle, EIS, lithium-ion batteries

## Abstract

Sodium-ion batteries (SIBs) have emerged as a promising alternative to lithium-ion batteries (LIBs) due to the abundant availability of sodium, lower costs, and comparable electrochemical performance characteristics. A thorough understanding of their performance features is essential for the widespread adoption and application of SIBs. Therefore, in this study, we investigate the output characteristics and electrochemical impedance spectroscopy (EIS) features of sodium-ion batteries (SIBs) under various states. The research results show that, unlike conventional lithium iron phosphate (LFP) batteries, SIBs exhibit a strong linear relationship between state of charge (SOC) and open-circuit voltage (OCV) across various SOC and temperature conditions. Additionally, the discharge capacity of the battery remains relatively stable within a temperature range of 15 °C to 35 °C; when the temperatures are outside this range, the available capacity of the sodium-ion battery reduces significantly. Moreover, the EIS profiles in the high-frequency region are predominantly influenced by the ohmic internal resistance, which remains largely unaffected by SOC variations. In contrast, the low-frequency region demonstrates a significant correlation between SOC and impedance, with higher SOC values resulting in reduced impedance, indicated by smaller semicircle radii in the EIS curves. This finds highlights that EIS profiling can effectively monitor SOC and state of health (SOH) in SIBs, offering a clear correlation between impedance parameters and the battery’s operational state. The research not only advances our understanding of the electrochemical properties of SIBs but also provides a valuable reference for the design and application of sodium-ion battery systems in various scenarios.

## 1. Introduction

Lithium-ion batteries (LIBs) have become indispensable across various energy storage applications, from storing energy generated by intermittent renewable sources to powering mobile devices and electric vehicles (EVs). However, the limited supply and high costs of key materials such as lithium, nickel, and cobalt present significant challenges. Even with advancements in battery recycling, the scarcity of these materials, particularly cobalt, raises concerns about the ability to meet the increasing global demand for energy storage solutions [1]. Sodium-ion batteries (SIBs) have emerged as a promising alternative to LIBs due to the abundant availability of sodium, lower costs, and comparable electrochemical performance characteristics. SIBs operate on a similar working principle as LIBs; however, the electrochemistry is governed by the intercalation and de-intercalation of sodium ions rather than lithium ions within the electrode materials during charging and discharging cycles. In addition, sodium salt raw material reserves are abundant and inexpensive, and with the use of iron-manganese nickel-based cathode materials compared to the cathode materials of LIBs, the cost of raw materials is reduced by half [2]; at the same time, due to the characteristics of the sodium salt, allowing the use of low-concentration electrolyte [2]. For this reason, SIBs are expected to replace conventional lead-acid batteries (LAB) and LIBs in large-scale energy storage due to their unique advantages in terms of cost.

The output characteristics of SIBs include normal voltage, available capacity, energy density, power density, cycle life, et al. These parameters serve as comprehensive indicators of an SIB’s stability and reliability. In addition, EIS is a powerful diagnostic tool that provides deep insights into the internal processes of batteries, including charge transfer resistances, ion diffusion, and the integrity of electrode materials [3]. By integrating the analysis of output characteristics and EIS profiles, we can assess battery performance, optimize design, enhance performance, diagnose faults, conduct maintenance, and evaluate materials [4,5]. To date, the output characteristics and the EIS profile evolution of sodium-ion batteries under different states remain insufficiently explored, so it is essential to study both the output characteristics and EIS profiles of SIBs under different states to advance our understanding and application of these batteries [6,7].

In order to improve the performance of SIBs, many scholars have conducted research, but most of the studies have been carried out from the perspective of the performance of SIB’s materials. For example, Zhao et al. [8] studied the output performance of SIBs using Na4C8H2O6 as a cathode material. Their results indicated that this material significantly reduces electrochemical polarization, achieving an average cell voltage of approximately 1.8 V. Komaba et al. [9] demonstrated that the reversible capacity and capacity retention of hard carbon anodes in SIBs are correlated with the battery voltage window. Wang et al. [10] investigated the use of graphene in SIBs, reporting a reversible capacity of up to 141 mAh g^−1^ at a current density of 40 mAg^−1^, with stability maintained over 1000 cycles. Ding et al. [11] explored different graphene structures for sodium-ion storage, finding that graphene carbonized at high temperatures exhibited larger interlayer spacing than conventional graphite. Kim et al. [12] reported that red phosphorus/carbon composites achieved a high reversible capacity of 1890 mAh g^−1^ and maintained a stable capacity of 1540 mAh g^−1^ at a high current density of 2.86 A g^−1^, attributed to the formation of the Na_3_P phase upon sodium insertion. Kang et al. [13] examined the performance of Co_9_S_8_/C composite anodes in SIBs, revealing that the electrochemical sodium storage performance of Co_9_S_8_/C significantly surpassed that of pure Co_9_S_8_ after 50 cycles. Jia et al. [14] employed electrospinning technology and post-annealing to embed ultrafine MoO_3_ into coal-derived carbon nanofibres. Cheng et al. [15] introduced a gel-based fumed silica electrolyte with methanol as an antifreeze additive, and results show that SIBs utilizing Na_2_SO_4_-SiO_2_ hydrogel electrolytes exhibited higher capacity retention and conductivity at low temperatures, primarily because the precipitation and growth of Na_2_SO_4_ grains are highly inhibited under these conditions. Zhang et al. [16] and Deng et al. [17] proposed the use of a novel two-dimensional (2D) buckled material and a three-dimensional (3D) mesoporous graphene oxide structure with the aim of enhancing the performance of sodium-ion batteries. Zhou et al. [18] developed a 2D NbSSe material as an anode for low-temperature SIBs, featuring a consolidated interlayer band gap and an optimized electronic structure facilitated by interlayer anionic ligands. To enhance the intrinsic long cycling performance and low-temperature sodium storage capabilities of SIBs, Wang et al. [19] significantly advanced a superior cathode material. Xing et al. [20] fabricated nanostructured cathodes based on a rationally designed N-hydroxy naphthalene imide sodium salt (NDI-ONa) for high-performance sodium-organic batteries, utilizing a bioinspired self-assembly strategy. This innovative approach aimed to enhance the electrochemical performance and stability of sodium-organic batteries. Peng et al. [21], a new Cd-based metal-organic framework has been constructed, which, as an anode material for LIBs, exhibited an initial discharge of 2486 mAhg^−1^ and a charge of 1683 mAhg^−1^ at a current density of 300 mAhg^−1^ with a high initial coulomb efficiency of 98%. Zhou et al. [22,23], studied nanoporous carbon nanomaterials with zeolite-type metal-organic skeletons as precursors and templates for lithium-ion batteries and furfuryl alcohol as the second precursor, and the results showed that the materials have good electrochemical properties and can be used as electrode materials for double-layer capacitors. In [24], an effective composite electrode design strategy of “assembly and phosphorization” is proposed to construct synergistic N-doped carbon-encapsulated NiCoP@N-C-based composites, employing metal-organic frameworks (MOFs) as sacrificial hosts.

Guo et al. [25] reported a high-voltage cathode composed of Na_3_V_2_(PO_4_)_2_O_2_F nano-tetraprisms (NVPF-NTP) to improve the energy density and low-temperature (LT) performance of SIBs. Their experimental results demonstrated that NVPF-NTP exhibits low strain (2.56% volume change) and favorable sodium transport kinetics during Na intercalation/deintercalation, offering long cycle life and excellent high-rate capability. Liu et al. [26] achieved the all-climate electrochemical performance of SIBs. Tian et al. [27] conducted studies about the carbonization mechanism of bituminous coal-derived carbon materials for lithium-ion and sodium-ion batteries. Wang et al. [28] proposed a specific electrolyte formulation comprising acyclic/cyclic ethers, which are thermally stable down to −150 °C and enable the formation of a stable solid electrolyte interphase (SEI).

Apart from that, some scholars have conducted research on estimation methods of state and reliability during the application of SIBs. For instance, a low-complexity and wide-adaptability data-driven model for SOC estimation of SIBs was proposed in [29]. The study indicated that the root mean square error (RMSE) for SOC estimation was 0.89% and 0.63% for 3.2 Ah and 10 Ah SIBs from different manufacturers, respectively. To improve the accuracy of SOC estimation for sodium and lithium-ion batteries, Sun et al. [30] proposed a model integrating deep learning methods. Experimental results showed that the fused model reduced RMSE by 11.24% and 74.44% compared to single N-BEATS and BiLSTM networks, respectively. Liu et al. [31] tested four commercial 26,700 SIBs at different SOCs and temperatures, then proposing an internal temperature estimation method based on EIS and machine learning (ML). Wang et al. [32] systematically discussed improvement strategies for enhancing SIB safety by analyzing the safety issues of different components, including electrolytes, anodes, and cathodes. Tyagaraj et al. [33] provided a comprehensive introduction to the charge storage mechanisms of SIBs and future development trends. Bai et al. [34] investigated the characteristics and challenges of low-temperature performance in sodium and lithium-ion batteries. Laufen et al. [35] presented a multi-method characterization of a commercial 1.2 Ah 18650 SIB.

Undoubtedly, the above studies have made great contributions to our in-depth understanding of the performance characteristics of SIBs. However, these studies have not fully considered the output characteristics and EIS variations of SIBs from the point of view of practical engineering applications. In fact, the output characteristics of the SIB are key to developing a BMS with higher performance and reliability. In addition, the EIS evolution of SIBs in different states can help understand the charge transfer resistance, ion diffusion, and other electrochemical phenomena of SIBs in practical engineering applications. By analyzing the EIS characteristics of SIB, it is possible to gain a deeper understanding of the degradation of battery performance and the failure mechanism. This enables the determination of an improvement path, which is the basis for the practical application and large-scale promotion of SIB engineering. Therefore, in this study, we will focus on the output characteristics and EIS evolution of the SIB in different states. By systematically analyzing these aspects under a variety of conditions, we aim to shed light on the factors affecting SIB performance and propose strategies to optimize SIB design and functionality, thereby providing valuable insights into improving SIB performance.

The structure of this paper is as follows. Section 2 explains the experimental setup and methodology used for output characteristics and EIS characteristics of SIBs. Section 3 presents the results of our analysis, highlighting key findings related to the performance of SIBs under different operational conditions, and discusses the implications of these results for the design and application of SIBs Section 4 concludes the paper with a summary of our findings and suggestions for future research directions. By addressing the current knowledge gaps in the understanding of sodium-ion battery performance, this research contributes to the ongoing efforts to develop cost-effective and efficient energy storage systems.

## 2. Test Rig for Sodium-Ion Batteries

### 2.1. Sodium-Ion Batteries

**Figure 1:**
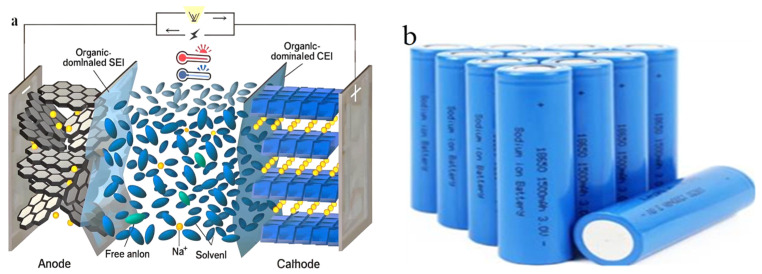
Principle of operation of SIB. (**a**) Schematic structure of sodium-ion battery (**b**) Sodium-ion battery samples.

Both sodium-ion and lithium-ion batteries consist of a positive electrode, a negative electrode, a diaphragm, and an electrolyte, which are very similar in structure, and both work by the migration of ions between the positive and negative electrodes to achieve charging and discharging. Unlike LIB, which is commonly used on the market today, SIB works by relying primarily on sodium ions moving between the positive and negative electrodes. The fundamental architecture of SIBs comprises a cathode and anode, typically separated by a non-aqueous electrolyte through which sodium ions shuttle back and forth (as shown in Figure 1). The cathodes are often composed of layered transition metal oxides, polyanionic compounds, or prussian blue analogs, which offer suitable sites for the storage of sodium ions. The anode materials range from carbon-based materials to alloys and metal oxides, each with distinct mechanisms for sodium storage. Among these, hard carbon has gained prominence owing to its favorable structural features that facilitate higher sodium storage capacity and promising cyclability. In SIBs, the structure and performance of the positive and negative materials determine the performance of the entire battery. The positive and negative electrodes are separated from each other by a diaphragm to prevent short-circuiting, the electrolyte infiltrates the positive and negative electrodes as a medium for ion circulation, and the collector plays the role of collecting and transferring electrons. During charging, Na^+^ is detached from the positive electrode and embedded in the negative electrode through the electrolyte through the diaphragm, so that the positive electrode is in a high potential sodium-poor state and the negative electrode is in a low potential sodium-rich state. The discharge process is the opposite, with Na+ coming out of the negative electrode and re-embedding into the positive electrode material via the electrolyte across the diaphragm, returning the positive electrode to a sodium-rich state. In order to maintain charge balance, the charge and discharge process have the same number of electrons through the external circuit transfer, with Na+ together in the positive and negative electrode migration, so that the positive and negative electrodes undergo oxidation and reduction reactions. It can be seen that the working principle of SIBs and rational ion batteries is basically similar and is also a class of “rocking chair battery”.

Due to the sodium ions being larger than lithium ions, their energy density is relatively low compared to LIBs. Despite this, recent advancements have resulted in SIBs achieving energy densities that are progressively closing the gap with their lithium-based counterparts, making them increasingly viable for a broad scope of applications, from grid storage to EVs. To better understand the output characteristics of SIBs and their EIS evolution law in different states, we conducted a detailed testing study, and the 18650 SIBs were chosen as the research object. The detailed parameters are shown in Table 1.

### 2.2. Experimental Details

**Figure 2:**
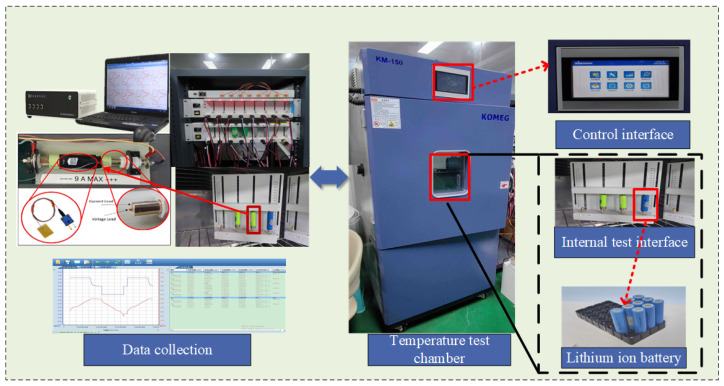
Test platform.

To accurately test the output characteristics and EIS of the SIB in a manner that reflects the conditions and scenarios encountered in real-world engineering applications, this section of the paper carries out an SIB testing study and improves the reliability and applicability of the results. All test parameters were set within the allowable operating range of the SIB during the testing process. The experimental test rig, designed for the study, is illustrated in Figure 2, and its specifications are provided in Table 2. It is important to note that this customized test rig is specifically configured to meet the unique requirements of SIB testing, allowing precise control and measurement of parameters such as charge/discharge currents, cut-off voltages, ambient temperatures, etc. The use of advanced data acquisition systems and real-time monitoring helps to capture key performance indicators and ensures the accuracy and fidelity of the recorded data so that the SIB’s behavior under different operating conditions can be fully analyzed. For the aging test of sodium-ion, we refer to the constant current and constant voltage (CC-CV) charging method of LIBs, which consists of a total of five stages, namely, constant-current charging, constant-voltage charging, stand, constant-current discharging, and stand or stop. The detailed implementation process is as follows: Step 1: Charge the batteries with a constant current of 1C until the terminal voltage of the batteries reaches the full charge voltage of 4.2 V; Step 2: Use a constant voltage (4.2 V) charging strategy to charge until the charging current drops to 50 mA, and then stop. Step 3: Let the batteries stand for 15 min. Step 4: Discharge the batteries with a constant current of 1C, and the cut-off voltage is 2.0 V. Step 5: Let the batteries stand for 15 min, and then go to the next cycle or stop the testing.

In Figure 2, the SIB test platform consists of a host computer, monitoring software, controllers, temperature chambers, electrochemical workstations, etc. The host computer serves as the central command unit, interfacing with the controller via the network port. This configuration permits the host computer to transmit commands and receive data from the control unit, thereby ensuring uninterrupted communication and data management. The temperature chamber is an integral component of the system and is specifically designed to replicate a range of operational conditions that the SIB may encounter. In addition, we can also conduct aging tests on this test platform that check the actual discharge capacity of the battery after each cycle is completed. The actual discharge capacity of the battery decreases as the number of cycles increases, and the SOH is defined as the ratio of the usable capacity to the initial state capacity. By precisely regulating the ambient temperature, the temperature chamber facilitates in-depth analysis of the thermal behavior and performance characteristics exhibited by the SIB.

The effectiveness of EIS measurements in characterizing SIB performance relies on accurate measurement of SIB impedance across a broad spectrum. In practical engineering applications, electrochemical workstations usually use current electrostatics and potential electrostatics to test the cell impedance spectrum waveform. Subsequently, the SIB charge transfer resistance, ion diffusion, and electrode material integrity in varying states can be analyzed based on the EIS profile, thus facilitating a more profound comprehension of the internal processes that regulate the performance of the SIB. This comprehensive setup not only enhances the reliability of the experimental results but also provides a robust foundation for evaluating the performance and reliability of SIBs across different operational conditions.

## 3. Results and Discussion

### 3.1. Preliminary Consistency Test

In order to ensure the accuracy of the experimental results and avoid the potential impact of any inconsistency in the SIB manufacturing process on the test results, we conducted a preliminary consistency test on the SIB samples before the start of the experimental study. The consistency tests encompassed the evaluation of parameters such as initial capacity, internal resistance, normal voltage, etc., thereby confirming that the selected batteries satisfy the requisite for this study. With this approach, the output characteristics and EIS curves of the SIB in different states can be evaluated more precisely, which helps to gain a more detailed understanding of its operational efficiency and reliability. The results of these consistency tests are presented in Table 3.

From Table 3, the SIB samples show remarkable consistency on all parameters, with the maximum error of available capacity being only 0.011 Ah (i.e., the sample SIBs with maximum and minimum available capacity are Bat. 4 and Bat. 3, respectively) and the maximum error of AC impedance being as small as 0.7 mΩ (i.e., the sample SIBs for maximum and minimum AC impedance are Bat. 2 and Bat. 4, respectively). The high consistency of key performance indicators not only validates the reliability of the selected samples but also improves the repeatability and accuracy of subsequent experimental results. Therefore, it can be concluded that these SIB samples have laid a good foundation for follow-up studies and provided a reliable basis for evaluating experimental results and drawing meaningful conclusions. In addition, the consistency of available capacity and AC impedance indicates stable electrochemical behavior, which is critical for analyzing output characteristics and EIS profiles under different operating conditions. This comprehensive preliminary validation helps to strengthen the rationality of the study design, thus ensuring that subsequent analyses of SIB performance are both accurate and reliable.

### 3.2. Open Circuit Voltage Testing

**Figure 3:**
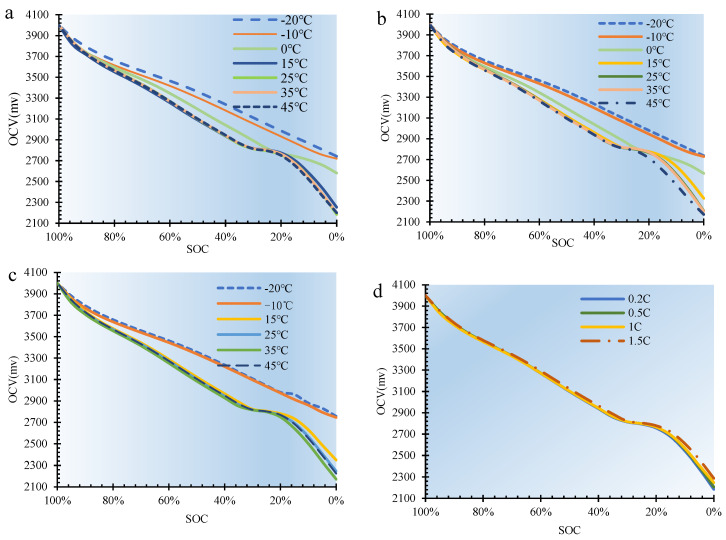
The SOC-OCV profiles of the SIBs. (**a**) When the battery is discharged at 0.1C (**b**) When the battery is discharged at 0.5C (**c**) When the battery is discharged at 1C (**d**) When the battery is discharged at 25 °C.

OCV testing is an essential technique for evaluating battery systems, not only to gain insight into the electrochemical state of the battery but also to estimate the SOC, providing indispensable data for the battery management system to ensure optimal operation and safety. In addition, the monitoring of OCV is of importance for the assessment of battery health, as any deviation from the standard OCV curve may indicate the presence of degradation or faults. In this section, the OCV of SIBs was measured within a temperature chamber, utilizing the methodology and test equipment described in Section 2, and the test results are shown in Figure 3. Figure 3a–c depicts the SOC-OCV profile obtained at different temperatures at a constant discharge current of 0.2C, 0.5C, and 1C, respectively. Figure 3d shows the SOC-OCV curves obtained at 0.2 C, 0.5 C, and 1 C discharge currents at a constant ambient temperature of 25 °C.

In Figure 3a, it is evident that the OCV of the SIB is significantly influenced by temperature regardless of the SOC. Specifically, a decrease in temperature corresponds to an increase in OCV. This behavior contrasts with that observed in lithium iron phosphate (LFP) batteries, as detailed in our previous studies [5]. Furthermore, as the SOC of the SIB decreases, its OCV generally follows a downward trend. Unlike conventional LFP batteries, SIBs exhibit less sensitivity to temperature changes within a certain range. For instance, in Figure 3a, at 40% SOC, the OCV at −20 °C is significantly different from the OCV at 45 °C. Additionally, there is a strong linear relationship between SOC and OCV across various SOC and temperature conditions. As the SOC decreases, the OCV declines linearly, in contrast to LFP batteries, which exhibit three distinct degradation stages: initial (100-95% SOC), stable (95-15% SOC), and rapid (below 15% SOC), as reported in our previous work [5]. Similar patterns are observed in Figure 3b,c. However, comparing Figure 3a–c reveals that the OCV is also dependent on the discharge current. Yet, within the 0.2C to 1.0C range, the current has minimal impact on the OCV curve of SIBs. For example, in Figure 3d, the OCV profile for SIBs at 25 °C under different discharge currents from 0.2C to 1C is nearly identical.

The available discharge capacity is a critical performance metric for SIBs. Existing research consistently indicates that LIBs, particularly lithium iron phosphate (LFP) batteries, are highly sensitive to temperature variations. At low temperatures, the discharge capacity significantly diminishes, with a marked decline observed when the temperature drops below 15 °C [5]. To investigate the impact of temperature on the performance of SIBs, we conducted tests at a discharge current of 1C. The available capacity of the SIBs was measured at different temperatures to understand the degradation behavior under varying operating conditions. The test results are presented in Table 4.

As evidenced by Table 4, the available capacity of SIBs is markedly affected by temperature. As temperature increases, battery performance improves, particularly at low temperatures, where capacity increase is more pronounced. For example, at a temperature of −20 °C, the available capacity of the SIBs is 800 mAh, whereas at 25 °C, the available capacity increases to approximately 1260 mAh, representing a 460 mAh increase. However, as the temperature continues to increase, the available capacity is observed to decline. For example, at 45 °C, a decline in capacity was observed, reaching 1209.5 mAh, which represents a reduction of 51.9 mAh in comparison to the capacity at 25 °C. Furthermore, the battery’s discharge capacity remains relatively stable when the temperature is above 15 °C. Although the performance tends to stabilize after the temperature exceeds 10 °C, it is still observed that the discharge capacity gradually increases with the temperature. This phenomenon can be attributed to the enhanced activity of electrochemical substances and materials within the battery, resulting in a higher discharge capacity.

It should be noted that the SOC in Figure 3 above is the ratio of the actual SOC in different temperature environments (both refer to Table 4), while in the testing process we set the voltage value of the initial state of the battery to a fixed value of 4.1 V. It is found that the initial discharge polarization phenomenon is not significantly affected by the temperature, but the findings also highlight the effect of temperature on the capacity decay of SIBs, underscoring the need to carefully consider temperature variations when predicting the RUL of SIBs. At the same time, it also provides an important reference for a comprehensive understanding of the relationship between temperature and battery performance and the development of more efficient and reliable energy storage systems.

### 3.3. EIS Testing

**Figure 4:**
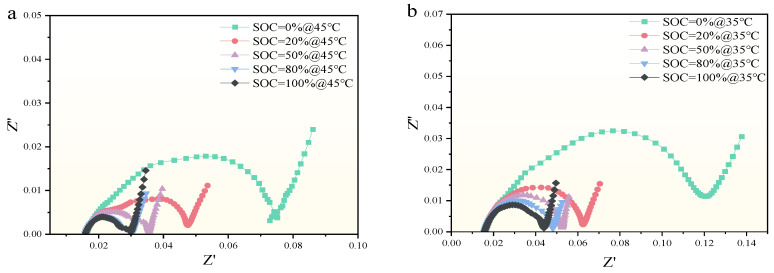

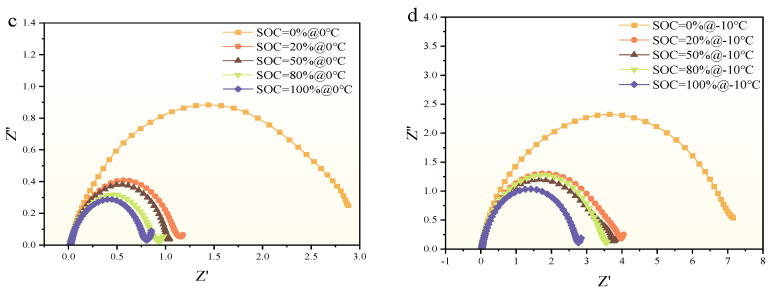
EIS characteristics of SIB under different SOC conditions. (**a**) EIS curves for different SOCs at 45 °C (**b**) EIS curves for different SOCs at 35 °C (**c**) EIS curves for different SOCs at 0 °C (**d**) EIS curves for different SOCs at −10 °C.

By using the electrochemical workstation depicted in Section 2, the EIS of the SIB was tested when it was at different SOCs and different temperatures. During the test, the SIB is scanned from 100 Hz to 10 kHz for each test. The test results are shown in Figure 4. Where Figure 4a–c shows the EIS profile obtained at different SOC and a constant discharge current of 45 °C, 35 °C, and 0 °C, respectively. Figure 4b shows the profile obtained when the temperature is constantly kept at −10 °C and with different SOC.

In Figure 4, it can be seen that the EIS profiles of SIBs are influenced by their SOC. The EIS profile shows varying degrees of change as the SOC values change. Each frequency region of the EIS profiles shows distinct characteristics under different SOC conditions, indicating significant differences in how SOC affects the EIS profiles of SIBs. Specifically, in the high-frequency region (HFR), the EIS profiles are nearly indistinguishable and tend to overlap. However, in the low-frequency region (LFR), the diffusion-related diagonal line of the EIS profile shows a more systematic variation with SOC; higher SOC values result in lower impedance values, and the semicircular arc shifts leftward as SOC increases.

At a constant temperature, the differences in EIS profiles across various SOC levels are more pronounced in the LFR. For example, in Figure 4a or Figure 4b, the semi-arc amplitude of the EIS profile is the largest when SOC is 0% at LFR and gradually decreases as SOC increases, while the amplitude is the smallest when SOC is 100%. In addition, the SOC range in SIBs affects the EIS profiles differently. For example, SOC values between 20% and 0% significantly alter the EIS profiles more than those between 20% and 50%. Moreover, from Figure 3, it is evident that SOC has minimal impact on the high-frequency EIS profiles, as these curves overlap significantly across SOC values of 100%, 80%, 50%, 20%, and 0%. This overlap indicates that lower SOC leads to increased impedance in SIBs. The findings suggest that the EIS profile in the LFR is particularly sensitive to SOC. Therefore, when using the EIS profile feature to estimate the SOC of SIBs, it is sufficient to focus on the low-frequency region EIS data rather than analyzing the entire EIS spectrum.

**Figure 5:**
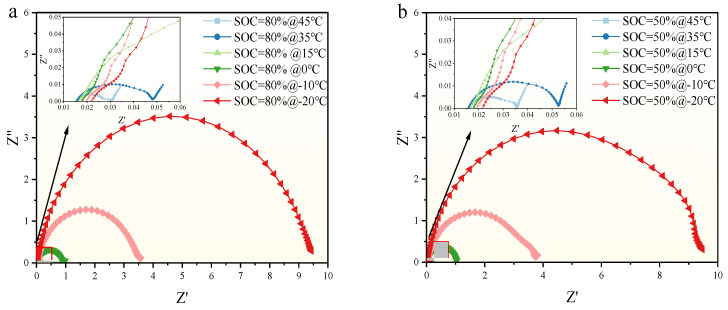
EIS characteristics of SIBs under different temperature conditions. (**a**) EIS curves for different temperatures at SOC 80% (**b**) EIS curves for different temperatures at SOC 50%.

To better illustrate the impact of EIS at different operating temperatures, Figure 5 presents the EIS profiles obtained at constant SOC values of 80% and 50% under different temperatures.

From Figure 5, we can see that the temperature significantly impacts the EIS of SIBs, with this effect being most pronounced at low temperatures. For instance, in Figure 5a,b, the EIS semicircular arc is largest at −20 °C, and its amplitude decreases progressively as the temperature increases. Furthermore, different temperature ranges affect the amplitude of the EIS semicircular arc to varying degrees. For example, the semicircular arc at −20 °C is approximately twice the size of that at −10 °C, and this disparity diminishes as the temperature rises. When the temperature exceeds 0 °C, the influence of temperature on the EIS semicircular arc under the same conditions gradually decreases. This indicates that under low-temperature conditions, it is crucial to consider the impact of temperature on SIBs.

**Figure 6:**
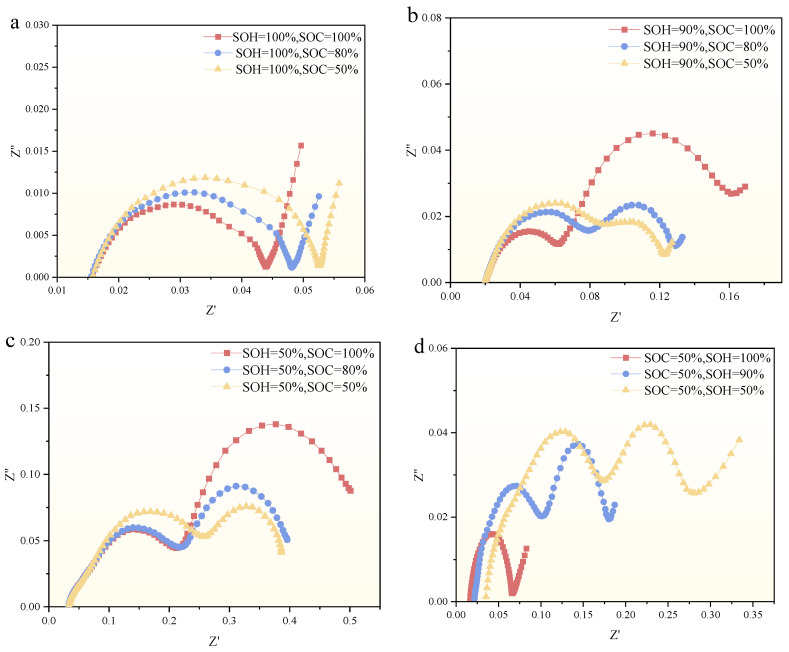
EIS profile of SIBs under different SOH. (**a**) EIS profile for different SOCs at 100% of the SOH (**b**) EIS profile for different SOCs at 90% of the SOH (**c**) EIS profile for different SOCs at 50% of the SOH (**d**) EIS profile for different SOHs at 50% of the SOC.

Figure 6 illustrates the EIS profile of SIB at various SOH and SOC states at room temperature (25 °C). From Figure 6, it can be observed that the EIS profile of SIBs is influenced by both SOH and SOC. Under the same SOH conditions, the change in EIS in the HFR is not affected by SOC; the EIS curves in this region are almost indistinguishable and overlap significantly. This indicates that the components of the ohmic internal resistance (e.g., diaphragm internal resistance, electrolyte internal resistance) are insensitive to changes of SOC. In contrast, as the test frequency decreases, the EIS profile of SIBs is more and more obviously affected by the SOC, and when the SOH is 100%, the larger SOC corresponds to the smaller EIS profile semicircular arc (as illustrated in Figure 6a). Furthermore, as illustrated in Figure 6, the EIS profile characteristics in different regions demonstrate that the SOC value has a negligible impact on the EIS curve profiles in the high-frequency region. As an illustration, when the SOH is 100%, 90%, and 50%, the profiles remain largely similar and overlap irrespective of the varying SOC statuses.

Apart from that, the change in SOH leads to a significant change in the EIS of the whole SIB, which is not only in the arc size but also in the curve shape, as shown in Figure 6d. It can be seen that as SOH increases (i.e., as the battery ages and degrades), the ohmic resistance (Ro) of the cell continuously rises, with the rate of increase accelerating significantly during the later stages of aging. This phenomenon can be attributed to the continuous formation and thickening of the solid electrolyte interface (SEI) film on the electrode surface throughout the aging process, which results in a relatively gradual increase in the battery’s ohmic internal resistance during the initial stages of aging. In addition, as SOH increased, the EIS profile showed significant changes over the entire frequency range (i.e., 10 KHz–100 Hz). For example, when the SOC was 100%, the EIS profile had only one peak point, while two peak points appeared when the SOH was 90 and 50%, which could be attributed to the fact that the internal electrochemical composition became more complex with the degradation of the SIB. Due to our limited experimental conditions, the exact reason may require more advanced equipment to carry out the study.

### 3.4. Discussion

The results of this study provide a comprehensive understanding of the output characteristics and EIS profile evolution of SIBs under various states. Therefore, we will discuss our findings in terms of output characteristics and EIS profile evolution of SIB, respectively.

#### 3.4.1. Output Characteristics

The analysis of the output characteristics revealed that there is a clear interaction between temperature, available capacity, SOC, and OCV. For example, at temperatures below 20 °C, the available capacity was observed to be significantly reduced, reaching a value of 800 mAh. This reduction can be attributed to the diminished ionic mobility and augmented internal resistance at lower temperatures, which impede the electrochemical reactions within the battery. As the temperature increased to 25 °C, a notable improvement in discharge capacity was observed, reaching 1260 mAh, an increase of 460 mAh. This improvement can be attributed to an enhanced rate of ion transport and a reduction in internal resistance, which facilitate more efficient electrochemical processes. However, an increase in temperature does not always promote an increase in the available capacity of the SIB, and when the temperature reaches a certain value, a continued increase in temperature can lead to a decrease in the available capacity of the SIB. For example, we found that further increases in temperature beyond 25 °C resulted in a slight decline in capacity, with a recorded value of 1209.5 mAh at 45 °C. This reduction can be attributed to the potential degradation of electrolyte stability and electrode material integrity at elevated temperatures, which may result in side reactions and a consequent reduction in the overall capacity. Apart from that, the OCV of the SIB is also significantly influenced by temperature, irrespective of the SOC. In particular, a reduction in temperature is associated with an increase in OCV. This behavior differs from that observed in LFP batteries, as detailed in previous studies [5]. In addition, as the SOC of the SIB decreases, its OCV generally follows a downward trend. In contrast to conventional LFP batteries, SIBs demonstrate a reduced sensitivity to temperature fluctuations within a defined range.

#### 3.4.2. EIS Profile Evolution

**Figure 7:**
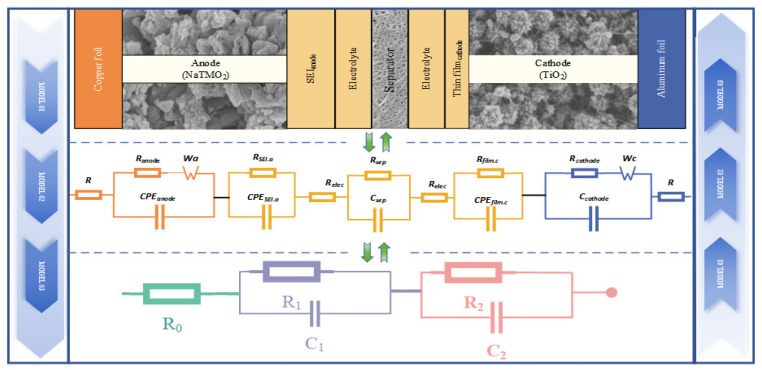
Structure schematic and equivalent circuit of LIBs.

The analysis of the impedance spectral evolution patterns of SIBs needs to be developed in conjunction with the corresponding equivalent circuit models, which usually equate the internal components of SIBs into multiple RC circuits. Each RC circuit in the model represents a polarization impedance, and theoretically, multiple RC circuits can describe the impedance values of different parts within a SIB, as shown in Figure 7. In practical applications, variations in electrode materials, as well as differences in the quality and surface area of the electrode materials, all contribute to differences in the EIS spectra. For this purpose, several RC circuits are required to characterize the performance of the battery by connecting them in series and parallel, which leads to more difficult calculations and does not allow research work to be carried out. To facilitate analysis, the multi-stage RC circuits are typically simplified into a second-order circuit model, as illustrated in Figure 7. Then, based on the test results and in combination with relevant software (e.g., Zview), the impedance values of these circuit elements can be fitted and obtained. This simplification helps in understanding the impedance characteristics and internal electrochemical processes more effectively.

In Figure 7, $R_{0}$ is the ohmic internal resistance, and it corresponds to the high-frequency region, with a frequency range of 10 kHz and above. $R_{1}$ is the internal resistance of the solid electrolyte interphase (SEI) at the interface between the electrode and the electrolyte. $C_{1}$ is the constant phase angle element of the SEI capacitive effect. $R_{2}$ is the internal resistance to charge transfer. $C_{2}$ is another constant phase angle element associated with the charge transfer process.

At lower temperatures (i.e., the temperature is −20 °C−0 °C), the EIS profile indicated elevated charge transfer resistance and diffusion impedance (shown in Figure 5), reflecting the impeded ion mobility and electrochemical reaction rates in the SIB, which coincides with the decrease in discharge capacity observed at lower temperatures in Table 4. With the increase in temperature, the EIS profiles showed a decrease in charge transfer resistance and diffusion impedance, indicating an increase in ion mobility and an increase in electrochemical reaction efficiency. This is why at low temperatures the impedance spectra of the semicircular amplitude of SIB show larger values in the LFR and overlap in the HFR, as shown in Figure 5a or Figure 5b. In addition, the EIS also shows the formation and stabilization of the SEI layer, and similar to LIBs, the SEI film in SIBs becomes progressively thicker as the charging and discharging cycles increase, leading to an increase in the corresponding ohmic impedance, as shown in Figure 6d.

#### 3.4.3. Practical Implications and Future Work

The findings of this study have significant implications for the design and optimization of SIBs for a range of applications. For example, the EIS feature can be effectively used to monitor the SOC and SOH of a SIB, and changes in impedance parameters with changes in SOC levels produce a clear correlation between electrochemical impedance and battery operating conditions. This correlation is important for the development of diagnostic instrumentation and management systems for SIBs to facilitate real-time monitoring and predictive maintenance. In addition, the sensitivity of discharge capacity to temperature highlights the need to implement an effective thermal management system to ensure optimal battery performance in a variety of environmental conditions. These findings are critical to establishing a reliable battery management system (BMS). The next step of the research will focus on improving the EIS analysis technique and investigating the effects of other environmental factors, such as humidity and mechanical stress, on the performance of SIBs, so as to provide reference experience for the design and development of BMS for SIBs as well as for market promotion and application.

## 4. Conclusions

The objective of this study was to conduct a comprehensive investigation into the output characteristics and evolution of the EIS profile of SIBs under a variety of operational conditions. The findings of this study can provide invaluable insights into the performance, reliability, and optimization of SIBs in practical engineering applications. The preceding work allows the following conclusions to be drawn.

(1) The discharge capacity of SIBs is highly sensitive to temperature variations, but the sensitivity of this effect varies across temperature intervals. For example, at low temperatures (−20 °C), the available capacity significantly decreases to 800 mAh due to reduced ionic mobility and increased internal resistance. As the temperature increases to 25 °C, the discharge capacity improves markedly to 1260 mAh, indicating enhanced ion transport kinetics and lower internal resistance. However, further temperature increases to 45 °C result in a slight decline in capacity to 1209.5 mAh.
(2) The OCV of SIBs is significantly influenced by temperature and SOC, and it is minimally affected by discharge current within the 0.2C to 1.0C range. A decrease in temperature leads to an increase in OCV and a strong linear relationship between SOC and OCV across various SOC and temperature conditions, which contrasts with the behavior observed in LFP batteries.
(3) The EIS profile of SIB exhibits alterations to varying degrees after different cycle times, influenced not only by material aging but also by the SOC and temperatures. The ohmic impedance of SIBs is largely unaffected by SOC, with the primary influence being the SOH. The main reason is that the internal ohmic resistance (Ro) of SIBs increases with aging due to the formation and thickening of the solid electrolyte interphase (SEI) film.

The aforementioned conclusions facilitate a deeper comprehension of the attributes of SIBs across diverse operational scenarios. Nevertheless, further investigation is imperative to consolidate this understanding. Given that sodium batteries represent a pioneering subject in our research domain, all the parameters established during the course of the tests were set in accordance with the recommendations provided by the manufacturer, particularly with regard to the permissible operating voltage range, capacity, and the charge/discharge currents for the batteries in question. Additionally, it was observed that the polarization phenomenon in the initial stage is less susceptible to temperature fluctuations when compared to that of a LiFePO4 battery. This phenomenon may be attributed to the battery material or the potential inaccuracy in the design of the battery parameters and performance parameters. These issues will be further investigated in subsequent research.

## Figures and Tables

**Table 1:** Parameters of SIBs.

Item	Value
Available capacity Ah	1.25
AC internal resistance (mΩ)	≤1.6
Weight (g)	40.1
Operating voltage range	4.2–2.0
Available temperature range for charging (°C)	0–45
Available temperature range for discharge (°C)	−20–50
Continuous charging/discharging current	1.5C
Nominal Voltage(V)	3.1

**Table 2:** Equipment parameters.

Specifications	Units	Values
Charging/Discharging equipment		
Measuring range of voltage	V	0–10
Measuring range of currents	A	0~20
Sample rate	Hz	1
Response time	mS	10
Potential increments	mV	0.1
Temperature chamber		
Adjustable temperature range	°C	−40–80
Temperature rise rate	°C/min	0.1
Volume of temperature chamber	L	60
Electrochemical workstation		
Measuring range of voltage	V	−10–10
Max. continuous current	mA	250
Bandwidths	MHz	1
Potential increments	mV	0.1
Minimum Sample Interval	us	1
Bias current	pA	≤10
Maximum sampling rate	MHz	1
Update rate	MHz	10
Max. data length	K	16,384
Accuracy of added potential	mV	±1
ACV Frequency Range	kHz	0.1–10

**Table 3:** Preliminary consistency test.

Item	Bat. 1	Bat. 2	Bat. 3	Bat. 4	Bat. 5	Bat. 6
Rated voltage (V)	3.12	3.11	3.11	3.09	3.13	3.11
Available capacity (Ah)	1.251	1.250	1.249	1.260	1.250	1.251
Charge capacity (Ah)	1.256	1.256	1.249	1.259	1.258	1.260
Weight (g)	40.23	40.23	40.13	40.33	40.22	40.24
AC impedance (mΩ)	15.1	15.5	15.4	14.8	14.9	15.3

**Table 4:** Available capacity of SIBs at different temperatures.

Item		−20 °C	−10 °C	0 °C	15 °C	25 °C	35 °C	45 °C
Capacity	Bat 1	800.5	820.7	1040.5	1180.5	1260.7	1200.3	1220.4
	Bat 2	800.8	822.2	1043.3	1182.7	1262.5	1201.0	1201.6
	Bat 3	800.7	821.8	1041.7	1181.8	1261.0	1201.3	1206.5
Average capacity (mAh)		800.7	821.6	1041.8	1181.6	1261.4	1200.9	1209.5

## Data Availability

Data will be made available on request.
